# Variable Lengths of Stay among Ischemic Stroke Subtypes in Chinese General Teaching Hospitals

**DOI:** 10.1371/journal.pone.0045101

**Published:** 2012-09-28

**Authors:** Yi Li, Hui Liu, Jing Wang, Yan Li, Guo-Pei Yu, Xie-Min Ma, Ming-Hui Liang, Jun Zhang, Lue Ping Zhao

**Affiliations:** 1 Peking University Medical Informatics Center, Peking University, Beijing, People’s Republic of China; 2 Department of Hospital Management of Peking University Health Science Center, Neurology Department of Peking University First Hospital, Peking University, Beijing, People’s Republic of China; 3 School of Public Health, Peking University Health Science Center, Peking University, Beijing, People’s Republic of China; 4 Institute of Hospital Management of the Ministry of Health, Beijing, People’s Republic of China; 5 Department of Hospital Management of Peking University Health Science Center, Neurology Department of Peking University Third Hospital, Peking University, Beijing, People’s Republic of China; Universidad Peruana Cayetano Heredia, Peru

## Abstract

**Background:**

Length of stay (LOS) is one of the most important quantitative indexes that measures health service utilization within a hospital. Many studies have examined the association of three major stroke categories with LOS. Our aim is to investigate the differences of LOS among ischemic stroke subtypes, results from which are helpful to healthcare providers and government agencies to improve health care delivery efficiency.

**Methodology/Principal Findings:**

Using the Beijing Municipal Health Bureau’s hospitalization summary reports, we performed a retrospective study among first-ever in-hospital patients with ischemic stroke (ICD-10 I63) in three general teaching hospitals in Beijing, China, from 2006 to 2010 with generalized linear model. In our study, 5,559 patients (female, 36.0%; age, 64.4±12.9 years) were included. The estimated mean LOS of ischemic stroke was 17.4±1.8 days. After adjusting for confounders, LOS of lacunar infarction (14.7 days; p<0.001) and LOS of small cerebral infarction (17.0 days; p = 0.393) were shorter than that of single cerebral infarction (17.9 days, p<0.001). LOS of multi-infarct (19.0 days; p = 0.028), brainstem infarction (19.3 days; p = 0.045), basal ganglia infarction (18.5 days; p = 0.452) and other subtypes of ischemic stroke (18.9 days; p = 0.327) were longer than that of single cerebral infarction.

**Conclusions:**

LOS of ischemic stroke patient differes across single cerebral infarction, lacunar infarction, multi-infarct and brainstem infarction patients. The ascending order of LOS was lacunar infarction, small cerebral infarction, single cerebral infarction, basal ganglia infarction, other subtypes of ischemic stroke, multi-infarct and brainstem infarction.

## Introduction

Stroke is the second most common cause of death and leading cause of adult disability worldwide [1]. Over two-thirds of stroke deaths worldwide are in developing countries [2]. In 2010, cerebrovascular disease ranks number 3 in China with a standardized mortality rate 143.54 per 100,000 behind cancers and heart diseases in urban areas and number 1 with standardized mortality rate 203.30 per 100,000 in rural areas [3]. In the United States, stroke ranks number 4 among all causes of death, behind the heart diseases, cancer, and chronic lower respiratory disease [4]. Because stroke often results in permanent dependence, long-term, cost-effective stroke care should be established [5], which inevitably leads to heavy use of health care resources. These resources are typically measured by the length of stay (LOS), which is probably one of the most important quantitative indexes that measures health service utilization within a hospital.

**Figure 1:**
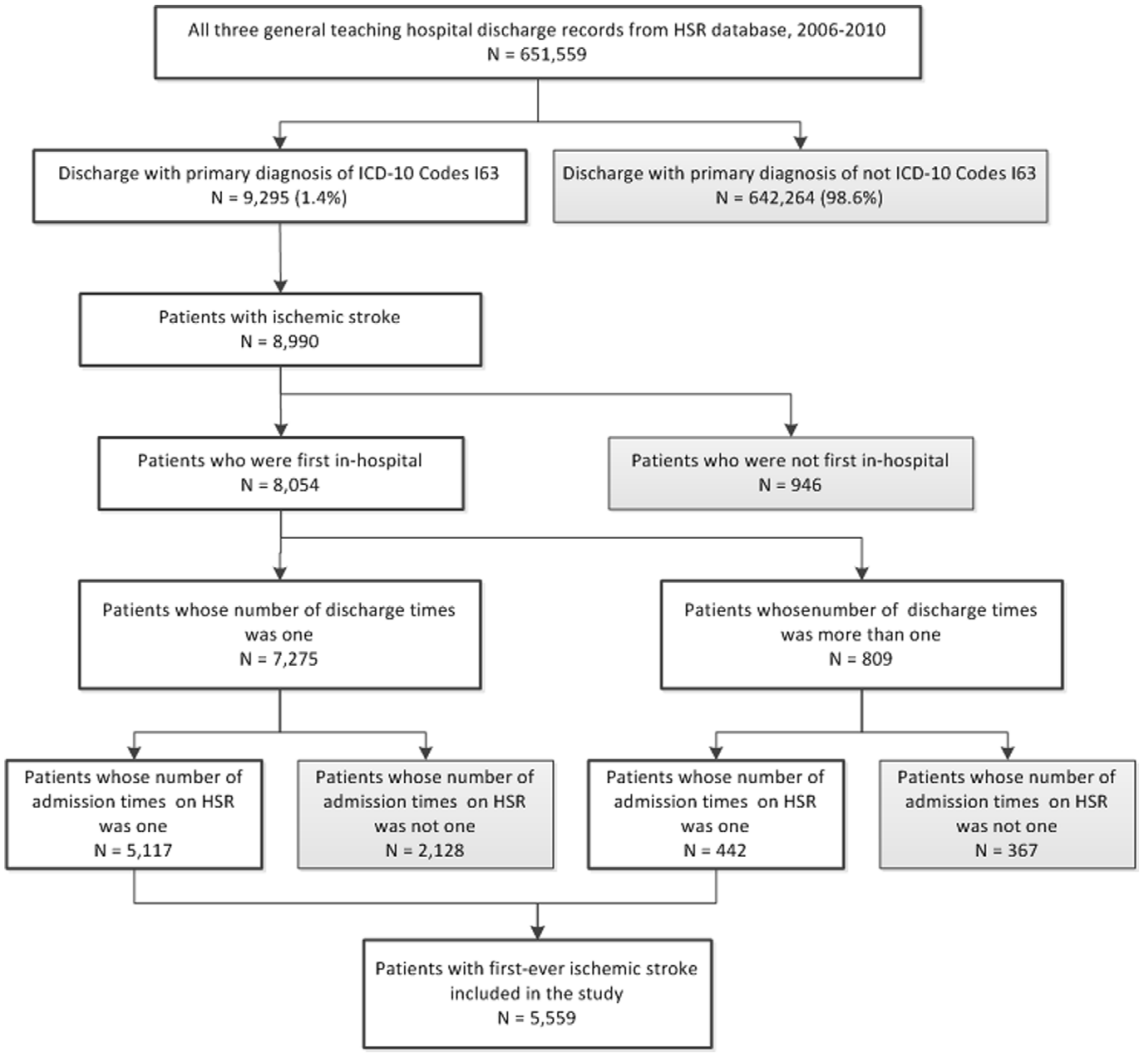
Graphic illustration of process of seleting first-ever ischemic stroke patients from the hospitalization summary report (HSR) database of Beijing Municipal Health Bureau, Beijing, China.

LOS of stroke is known to associate with many demographic and clinical factors, and observed associations are sometimes inherent within health care systems. In Belgium, for example, LOS has been shown to depend on disease severity at entry, location where the stroke strikes, and social economic status [6]. Also, there are reports concerning association of stroke and LOS, most of which concern three major stroke categories (ischemic stroke, 87%; intracerebral hemorrhagic stroke, 10%; and subarachnoid hemorrhage stroke, 3%) [4], [7]–[13]. To the best of our knowledge, few studies have been conducted to examine the association of ischemic stroke subtype with LOS, in light of multiple factors that may influence LOS. Given surged interest in using LOS to evaluate hospital performance and to determine financial payment, it seems important and necessary to examine variations of LOS across subtypes. The aim of this study is to gain insight into contributing factors to LOS in an unbiased fashion, thus more accurately providing concrete evidences for health care providers and government to improve healthcare delivery strategies, as China is undergoing modernization. Besides informing international communities, this manuscript also hopes to share the China experience with other developing countries as they work to improve their health care delivery systems.

**Figure 2:**
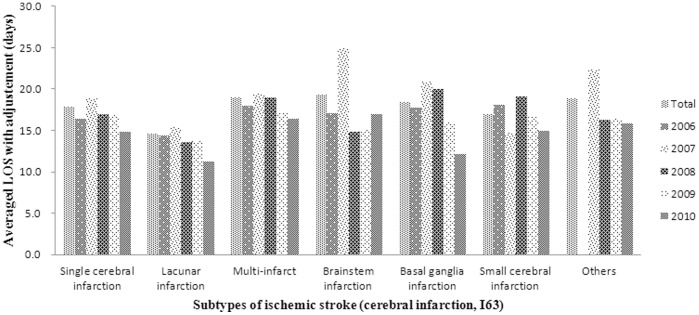
Averaged LOS of ischemic stroke subtypes with adjustment for confounding variables during five years (2006–2010). There was no average LOS value for other subtypes of ischemic stroke in 2006, because the number of other subtypes of ischemic stroke patient was zero.

## Methods

All data used in this study were extracted from the hospitalization summary report (HSR) database of the Beijing Municipal Health Bureau, Beijing, China. By governmental mandate, HSR on each hospitalization is required as a routine report to Beijing Municipal Health Bureau. The information assists the Bureau to make health care-related decisions, to allocate financial resources and to monitor health care services. Given the critical importance of these decisions, the Bureau, since 2004, has been consistently improving HSR reporting quality through standardizing the reporting form and enforcing the reporting procedure [14].

### Study Population

We chose three general teaching hospitals, affiliated with Peking University, and all patients were hospitalized with first-ever ischemic stroke as primary diagnosis in these hospitals during year 2006 and 2010. Head plain CT or MRI examination was carried out on all suspected stroke patients and head plain CT scan before thrombolytic treatment was also carried out according to the guidelines of ischemic stroke diagnosis and treatment [15], [16]. Examination results are recorded in electronic medical records, but are not routinely reported on HSR. These three hospitals are comparable with respect to number of hospital beds (1,500, 1,448 and 1,284).

### ICD-10 Classification of Ischemic Stroke

The codes for stroke (cerebrovascular disease) were I60 to I69 in International Classification of Diseases, 10th Revision (ICD-10) [4]. ICD-10 codes for acute ischemic stroke (AIS) include H34.1 (central retina artery occlusion), I63.x (cerebral infarction) and I64.x (stroke, not specified as hemorrhage or infarction) [9]. According to the classification standard of Beijing Municipal Health Bereau, ischemic stroke was classified to cerebral infarction (I63).

Focusing on cerebral infarction, we used the six digit code of disease diagnosis modified by Beijing Municipal Health Bereau based on ICD-10 (from I63.301 to I63.909) [17]. For subordinate diagnosis of patients with cerebral infarction, we referenced both the three digit code and the six digit code of ICD-10 (from A00 to Z99).

### LOS

LOS was defined as the difference between admission to discharge, death or other residential institution. Numerically, it was calculated by discharge date minus admission date. If admission date and discharge date were the same, LOS was set to 1 day [10]. Given the primary focus on LOS, the study analysis treated LOS as a dependent variable throughout all analyses.

### Ischemic Stroke Subtypes

The different primary diagnoses of cerebral infarction, reported on HSR, were used to represent the subtypes of ischemic stroke. Given the primary focus on association of LOS with ischemic stroke subtype, this variable was treated as the primary independent variable through all analyses.

### Confounding Variables

Just as any typical clinical studies, patients who receive hospitalization vary widely, some of which may confound the association analyses of interest. We considered the following potentially confounding variables: gender is potentially a confounder, since the stroke incidence differs between males and females, and so are hospitalization rates [18]. Age at admission by admission date minused birth date as a confounder was categorized according to commonly used in related researches [19]. Another important confounder is payment methods, which can be largely categorized into at least five types: social basic medical insurance, commercial insurance, out-of-pocket, governmental, comprehensive arrangement for serious disease, and other payment methods. Hospital and discharge year were also taken as confounders. Because comorbidity and complication were associated with large increases in the LOS [20], [21], subordinate diagnosis is another potential confounding variables in our study. Finally, three additional potential confounders are disease status at admission (critical: refers to patients with unstable vital signs, and a in direct threat to the lives of patients, requiring immediate rescue; serious: refers to patients with acute disease, acute exacerbation of chronic disease, acute poisoning or accidental injury, requiring immediately confirm the diagnosis and treatment; general: situation other than the critically ill and emergency serious), treatment method (western medicine with or without either traditional Chinese patent medicine or traditional Chinese herbal medicine [16]), and primary outcomes at the discharge (recovered: refers that disease symptoms is eliminated and the function is fully restored after treatment; improved: refers that disease symptoms is alleviated and the function is partly restored after treatment; no improvement: refers that the disease has no amelioration or deterioration; death: refers to those who died after hospital admission; others: refers to the person discharginges without treatment).

### Statistical Analysis

Throughout all analyses, LOS was treated as the primary response variable. To ensure appropriate statistical properties, we visually examined the distribution of LOS with computed the kurtosis and skewness, which appears to have an extreme long tail on the right hand side of the distributions. Our exploration suggested that logarithmic transformation seemed to correct this skewed distribution. The resulted value was referred to as logarithmic length of stay (LLOS). Besides computing arithmetic mean and median of LOS, we also used anti-logarithmic method to compute geometric mean of LOS and related 95% confidence intervals (CI). All tests were 2-tailed with significance at 0.05.

The generalized linear model (GLM) was used to assess relationship of LLOS with ischemic stroke subtypes. GLM is a flexible statistical model that is applicable to various types of outcome variables. To gain an overall impression on this relationship, we chose to perform an unadjusted analysis with the GLM. To adjust all of potential confounding variables, we used GLM again to evaluate the associations of interest. In GLM Univariate, the LLOS was treated as outcome, whereas the subtypes of ischemic stroke were taken as dummy indicators in the regression. For the adjusted analysis, GLM includes not only those dummy indicators of ischemic stroke subtypes, but also all possible confounders as covariates.

The statistical package, SPSS (Version 17.0), was used to perform all of analyses.

## Results

This study included a total of 5,559 patients from three general teaching hospitals, each of which contributes 1,854, 2,160 and 1,545 patients, respectively. The process used to select first-ever ischemic stroke patients is described through Figure 1. Table 1 summarizes the patient’s characteristics: gender, age, payment method, hospital, discharge time, severity at admission, secondary diagnosis and outcome at discharge. There were more male patients (64%) than female patients (36%). The mean age of all patients at admission was 64.4±12.9 years, with 91.3% of patients between 45 to 84 years old. With respect to payments, it appeared that out-of-pocket payment was the most common payment method (38.4%), followed by basic medical insurance (37.9%) and governmental payment (18.1%). Across five years, frequencies of first-ever hospitalizations were around 20% per year. 38.7% of patients were classified to be in critical or serious status. There were 18,442 subordinate diagnoses for 5,559 patients, among whom only 206 (3.7%) had no subordinate diagnosis. Among all the 18,442 subordinate diagnoses, top 10 diseases were hypertension (3,845, 20.9%), type II diabetes (1,691, 9.2%), hyperlipidaemia (1,178, 6.4%), infection (982, 5.3%, included pneumonia, respiratory infections, intestinal infection, urinary tract infection and septicemia, etc.), arteriosclerosis (773, 4.2%), coronary artery disease (618, 3.4%), fatty liver (485, 2.6%), cervical spondylosis (417, 2.3%), atrial fibrillation and atrial flutter (386, 2.1%) and cerebral infarction sequela (298, 1.6%). Few patients died during hospitalization (2.0%), and the majority was reported to have improvement (93.3%) or complete recovery (3.2%), with 1.0% of patients reported to have no improvement.

**Table 1:** Distributions of gender, age, payment method, hospital, discharge year, subordinate diagnosis, severity at admission, treatment method and primary outcome at discharge for 5,559 patients.

Variable	Frequency	%
**Gender**		
Male#	3,559	64.0
Female	2,000	36.0
**Age at Admission (**year)		
< = 34	84	1.5
35–44	235	4.2
45–54	1,015	18.3
55–64	1,274	22.9
65–74#	1,608	28.9
75–84	1,178	21.2
> = 85	165	3.0
**Payment Method**		
Basic medical insurance	2,109	37.9
Out-of-pocket payment#	2,133	38.4
Governmental payment	1,006	18.1
Others	311	5.6
**Hospital**		
Hospital 1	1,854	33.4
Hospital 2#	2,160	38.9
Hospital 3	1,545	27.8
**Discharge Year**		
2006	1,017	18.3
2007	1,138	20.5
2008#	1,155	20.8
2009	1,134	20.4
2010	1,115	20.1
**Subordinate Diagnosis**		
Not infectious, with hypertension, type II diabetes or hyperlipemia#	3,874	69.7
Not infectious, without hypertension, type II diabetes or hyperlipemia	883	15.9
Infectious, with hypertension, type II diabetes or hyperlipemia	627	11.3
Infectious, without hypertension, type II diabetes or hyperlipemia	175	3.1
**Severity at Admission**		
Severe or critical	2,152	38.7
General#	3,407	61.3
**Treatment Methods**		
Western Medicine and TCPM* ^#^	2,651	47.7%
Western Medicine	2,219	39.9%
Western Medicine, TCPM and TCHM**	478	8.6%
Western Medicine and TCHM	204	3.7%
Others	7	0.1%
**Outcome at Discharge**		
Recovered	180	3.2
Improved#	5,187	93.3
No improvement	58	1.0
Death	110	2.0
Others	24	0.4

* TCPM  =  Traditional Chinese Patent Medicine.

** TCHM  =  Traditional Chinese Herbal Medicine.

# Dummy variables taken as references in parameter estimate of GLM Univariate.

The frequency and percent of the 5,559 patients by ischematic stroke subtype in our study are listed in Table 2. Originally, I63.902 was cerebral infarction. In order to distinguish with the class heading of I63, we used single cerebral infarction instead of cerebral infarction to express the meaning of Not Multi-infarct and Not Otherwise Specified (NOS). Based upon frequencies, we combined these sixteen subtypes into seven subtypes. Single cerebral infarction was most common (75%), followed by lacunar infarction, multi-infarct, brainstem infarction, basal ganglia infarction, small cerebral infarction and other combined.

**Table 2:** ICD-10 Codes and names of cerebral infarction and their frequency (N) and percentage (%) among all cases diagnosed in this study.

Code	Name	N	%
I63	Cerebral infarction		
I63.301	Cerebral thrombotic hemiplegia	0	0.0
I63.302	Cerebral infarction due to thrombosis of arteries	5	0.1
I63.401	Cerebral embolic hemiplegia	0	0.0
I63.402	Cerebral infarction due to embolism of arteries	15	0.3
I63.501	Cerebral infarction due to occlusion of arteries	4	0.1
I63.502	Cerebral infarction due to stenosis of arteries	13	0.2
I63.601	Cerebral infarction due to cerebral venous thrombosis, non-pyogenic	0	0.0
I63.901	Multi-infarct	394	7.1
I63.902	Single cerebral infarction	4,181	75.2
I63.903	Lacunar infarction	502	9.0
I63.904	Small cerebral infarction	66	1.2
I63.905	Hemorrhagic infarction	33	0.6
I63.906	Brainstem infarction	188	3.4
I63.907	Basal ganglia infarction	138	2.5
I63.908	Carotid artery occlusion	9	0.2
I63.909	Vertebral artery occlusion	11	0.2
Total		5,559	100.0

Next we explored the distributions of LOS and LLOS (Table 3) across seven ischemic stroke subtypes. Within each subtype, we computed arithmetic means, standard deviations (SD) and medians on LOS on the left hand panel, along with their kurtosis and skewnesses. Clearly, both kurtosis and skewness indicated departure from the desired Gausian distribution (kurtosis and skewness of normal distribution: 3, 0). Further, the mean values were much greater than those median values across all seven groups. In contrast, kurtosis and skewness statistics on the LLOS (the right panel of Table 3) indicated that LLOS approximately had a normal distribution and hence had desired statistical properties. By taking anti-logarithmic transformation to LLOS, we obtained estimated mean LOS, which were closer to median values as expected.

**Table 3:** Estimated LOS by arithmetic means and median, averaged logarithmic LOS (LLOS) with its standard deviations, kurtosis and skewness across seven subtypes of ischemic stroke.

	N	LOS				LLOS			
		Mean±SD (day)	Median (day)	Kurtosis	Skewness	Mean±SD	estimated LOS SD (day)	Kurtosis	Skewness
Single cerebral infarction	4,181	20.3±21.1	17	1,811.9	35.2	2.9±0.5	17.4±1.7	4.2	−0.5
Lacunar infarction	502	15.9±7.3	14	5.0	1.6	2.7±0.5	14.301.6	2.0	−0.5
Multi-infarct	394	23.7±16.3	20	10.7	2.8	3.0±0.6	20.0±1.8	1.2	0.2
Brainstem infarction	188	33.6±99.7	22	161.4	12.4	3.0±0.8	21.2±2.2	6.5	−0.1
Basal ganglia infarction	138	23.9±31.2	20	104.8	9.7	3.0±0.5	19.5±1.7	5.7	1.0
Small cerebral infarction	66	21.1±11.2	19	1.7	1.1	2.9±0.7	17.8±2.0	7.9	−2.1
Others	90	22.1±14.5	19	8.8	2.3	2.9±0.7	18.3±1.9	1.3	−0.6
**Total**	5,559	20.7±27.0	17	1,542.5	34.4	2.9±0.6	17.401.8	4.5	−0.3

As a formal analysis on the association of LLOS with the subtypes, we used the GLM to estimate means of LLOS on indicators of all subtypes (Table 4). For these analyses, we treated single cerebral infarction as the reference subtype to compare it with the other subtypes. Without adjusting for possible confounders, LOS of lacunar infarction was significantly shorter than that of single cerebral infarction (14.3 versus 17.3 days, p-value <0.001). On the other hand, LOS of multi-infarct and brainstem infarction, basal ganglia infarction were significantly longer than that of single cerebral infarction (19.8 versus 17.3 days, p-value <0.001; 20.8 versus 17.3 days, p-value <0.001; and 19.3 versus 17.3 days, p-value = 0.015, respectively). It appeared that the LOS of small cerebral infarction was comparable to that of the reference subtype (17.7 versus 17.3 days, p-value = 0.720), and other subtypes combined were not significantly different from the reference subtype (18.2 versus 17.3 days, p-value = 0.395). Adjusting for all possible confounders listed above, the right panel of Table 4 listed LOS mean, its SD, estimated LLOS, 95% CI, LOS differences from the reference subtype (D-value), and p-values. Clearly, LOS of lacunar infarction remained significantly different from that of single cerebral infarction (14.7 versus 17.9 days, p-value <0.001), and as did the LOS of multi-infarct and brainstem infarction (19.0 days, p-value = 0.028; and 19.3 days, p-value = 0.045, respectively). Interestingly, corresponding LOS estimates for later two subtypes were shorter by nearly one day, i.e., from 19.8 to 19.0 days and from 20.8 to 19.3 days, respectively, mostly due to confounding effects. After adjusting these confounders, the difference of LOS between basal ganglia infarction and the reference subtype was almost eliminated (0.6 days, p-value = 0.626).

**Table 4:** Estimated marginal means and related statistics for assessing associations of ischemic stroke subtype with LLOS, without and with adjusting for all confounding variables by univariate of general linear model.

	Un-adjusted							Adjusted						
	LLOS Mean	Std. Error	estimated LOS Mean (day)	95% CI		D-value (day)	p- value	LLOS Mean	Std. Error	estimated LOS Mean (day)	95% CI		D-value (day)	p- value
				Lower Bound	Upper Bound						Lower Bound	Upper Bound		
Single cerebral infarction*	2.854	0.009	17.3	17.0	17.6		0.000	2.868	0.008	17.5	17.3	17.8		0.000
Lacunar infarction	2.662	0.025	14.3	13.6	15.0	−3.0	0.000	2.667	0.024	14.4	13.7	15.1	−3.1	0.000
Multi-infarct	2.997	0.028	19.8	18.8	20.8	2.5	0.000	2.932	0.027	18.6	17.7	19.5	1.1	0.021
Brainstem infarction	3.052	0.040	20.8	19.3	22.3	3.5	0.000	2.953	0.039	19.0	17.7	20.3	1.5	0.033
Basal ganglia infarction	2.972	0.047	19.3	17.7	21.0	2.0	0.015	2.881	0.045	17.7	16.3	19.2	0.2	0.778
Small cerebral infarction	2.879	0.068	17.7	15.6	20.0	0.4	0.720	2.847	0.064	17.2	15.2	19.3	−0.3	0.745
Others	2.905	0.059	18.2	16.3	20.2	0.9	0.395	2.912	0.055	18.3	16.5	20.2	0.8	0.426

* Single cerebral infarction was taken as basement of D-value.

Between 2006 and 2010, the total estimated mean LOS of ischemic stroke decreased from 18.2 to 16.3 days. The unadjusted estimated mean LOS of single cerebral infarction, lacunar infarction, multi-infarct, basal ganglia infarction and small cerebral infarction decreased over time by 10.3%, 8.6%, 26.1%, 4.4%, 34.0%, and 13.6% each year, respectively, but that of brainstem infarction increased by 6.6%. The adjusted estimated mean LOSs of single cerebral infarction, lacunar infarction, multi-infarct, basal ganglia infarction and small cerebral infarction decreased by 9.6%, 21.5%, 8.7%, 31.4%, and 17.5%, respectively, while that of brainstem infarction decreased by 0.3% (Figure 2).

## Discussion

In China, clinical epidemiological studies of neurological diseases began in the early 1980s. Given fast changing health care environment, updated and accurate data on stroke hospitalization are needed to help planning strategies on prevention and management of stroke [22]. LOS has been advocated to be an important indicator for the efficiency of delivering hospitalization care. Often, an overall LOS of an entire hospital has been used as a measure of hospital efficiency, without acknowledging variations of LOS across different diseases or case mix. Even within a single disease, such as ischemic stroke, LOS could also vary across stroke subtypes, which has been shown in this manuscript. Specifically, LOS could vary from 14.7 days for lacunar infarction to 19.3 days for brainstem infarction, a difference of 4.6 days. This recognition is important for health care management branches to take into account when evaluating hospital performance, as well as for hospital management to allocate sufficient hospital resources in accordance to distributions of ischemic stroke subtypes.

The analysis presented above showed that the average LOS of ischemic stroke was around 17.4 days and that this LOS was greater than reported LOS from other hospitals in China. For example, in 2010, it was reported that the LOS for cerebral infarction in the general hospital in cities was 13.6 days, and that of intracranial hemorrhage was 15.1 days [23]. While it is possible that efficiency in other hospitals is higher, there are other explanations for these discrepancies. One plausible reason is the referral bias of patients; being top teaching hospitals, the three hospitals under consideration are among the best hospitals in Beijing and likely receive patients with more severe and complicated conditions. Further, these three teaching hospitals may provide more services than other hospitals, including not only necessary acute treatment but also rehabilitation immediately following the treatment. Indeed, integrated stroke care has been shown to provide better clinical outcomes than conventional care, probably with longer LOS [7], [24]. An earlier study has shown that the average LOS was about 4 days longer with integrated care in the stroke unit (19 versus 15 days) [9].

Estimated LOS across all subtypes was around 17.4 days, much greater than LOS in comparable teaching hospitals from the United States. It is known that the mean LOS associated with ischemic stroke reported from academic medical centers within a University Health System Consortium was 7.7 days [25], while the median LOS of academic centers was around 6 days [26] and mean LOS of teaching hospital was 10.8 days [27]. The primary reason for this discrepancy is associated with the fact that stroke treatment and post-treatment rehabilitation are well separated in the United States, and hence the time in rehabilitation is typically not included in the calculation of LOS. However, we should not exclude the possibility that physicians in the United States may have much more effective treatment strategies in hand than Chinese physicians; the higher quality of care during the early phase of stroke was associated with shorter LOS among patients with stroke [28]. This possibility, which can not be confirmed with the available data, should encourage Chinese neurological specialists to study carefully the typical clinical pathways in the standard care of stroke patients in United States, the knowledge from which may improve our treatment efficiency. Fortunately, LOS across all subtypes have been steadily declining over these five years, demonstrating the continuing improvement in hospitalization efficiency.

While this study is probably among the largest studies on LOS of stroke, we need to be cognizant of the potential limitations with HSR as the primary data source. Some patients may have multiple episodes of strokes, and hence have multiple hospitalization records. To eliminate potential biases associated with these patients, we considered only first-ever stroke patients. However, from the HSR data, we were not able to eliminate those patients who may have sought care prior to coming one of these teaching hospitals, but number of such patients was probably small. Since HSR is used for routine reporting to the Beijing Municipal Health Bureau, it does not provide sufficient details to separate complications arising from the current episode of the stroke from the comorbidity of the disease. Hence, the clinical variable on subordinate diagnosis should be interpreted as either complication or comorbidity. Finally, there may be coding errors or inconsistencies within ICD-10 with local modifications. An effort has been made to ensure consistent ICD-10 coding across three hospitals. However, we do not have a direct access to electronic medical records (EMRs) to determine the accuracy of ICD-10 coding on HSR at this time. However, mis-coding of stroke subtypes typically attenuates the association analysis to the null. Hence, the true associations would be even stronger than those observed here; i.e., estimated LOS could have even greater differences than those observed here. Nevertheless, further evaluation based on EMRs may be helpful, in minimizing error associated with incorrect ICD-10 coding.

In conclusion, we found that LOS of ischemic stroke patients differes significantly across single cerebral infaction, lacunar infarction, multi-infarct and brainstem infarction patients. The ascending order of LOS was lacunar infarction, small cerebral infarction, single cerebral infarction, basal ganglia infarction, other subtypes of ischemic stroke, multi-infarct and brainstem infarction. This result could help hospitals to anticipate usage of hospital resources, and also to motivate investigation of clinical pathways associated with subtypes of ischemic stroke in order to improve clinical care and shorten LOS.
